# Severe Respiratory Syncytial Virus Bronchiolitis Complicated by Bilateral Massive Pleural Effusions in a Previously Healthy Infant: A Case Report

**DOI:** 10.7759/cureus.108167

**Published:** 2026-05-03

**Authors:** Fadl Abdulaal, Dima Bani Issa, Dua' N Samara, Loay Abdulsamad, Fadi Toonsi

**Affiliations:** 1 Department of Neonatology, Dr Sulaiman Al Habib Medical Group (HMG), Jeddah, SAU; 2 Pediatric Intensive Care Unit, Dr Sulaiman Al Habib Medical Group (HMG), Jeddah, SAU; 3 Department of Pediatric Pulmonology, University of Colorado Anschutz Medical Campus, Aurora, USA; 4 Pediatric Intensive Care Unit, King Faisal Specialist Hospital and Research Centre, Jeddah, SAU; 5 Department of Radiology, King Abdulaziz University, Jeddah, SAU

**Keywords:** bronchiolitis, mechanical ventilation, pleural effusion, respiratory failure, rsv

## Abstract

Respiratory syncytial virus (RSV) bronchiolitis is one of the leading causes of hospitalization in infants, typically presenting with mild-to-moderate respiratory distress. Severe complications such as apnea, respiratory failure, and pneumothorax are known, but massive pleural effusions remain an exceptionally rare manifestation.

We report a case of an eight-week-old previously healthy Saudi female infant presenting with severe RSV bronchiolitis. The patient developed progressive respiratory failure requiring mechanical ventilation, re-intubation, and circulatory support. Notably, she developed bilateral massive pleural effusions necessitating urgent bilateral pigtail catheter insertion. Pleural fluid was sterile and transudative in nature. The infant showed significant clinical improvement following drainage and supportive care, with eventual full recovery.

This case highlights an unusual but severe complication of RSV bronchiolitis. It underscores the need for clinicians to consider pleural effusion in infants with RSV who deteriorate despite supportive measures, even in the absence of bacterial infection.

## Introduction

Respiratory syncytial virus (RSV) bronchiolitis is one of the leading causes of hospitalization in infants, accounting for a substantial proportion of acute lower respiratory tract infections and an estimated 3.6 million RSV-associated hospitalizations in children under five years of age worldwide each year [1]. Most infants experience a self-limited course, although severe complications such as respiratory failure, apnea, and the need for escalating respiratory support are well described, particularly in those with underlying comorbidities [2,3].

RSV bronchiolitis primarily affects the small airways, and significant pleural involvement is not a typical feature of viral lower respiratory tract infections. Pleural effusion in this context is therefore considered uncommon, with only a limited number of cases reported in the literature. Reports of large, bilateral pleural effusions in the absence of bacterial superinfection are particularly rare [4-6].

We report a case of an otherwise healthy infant with severe RSV bronchiolitis who developed large bilateral pleural effusions without evidence of bacterial infection. This case highlights an unusual and poorly characterized complication and aims to contribute to the understanding of potential inflammatory and mechanical mechanisms in viral-induced pleural effusions.

## Case presentation

An eight‐week‐old Saudi female infant, who was born at term (birth weight ~2.5 kg) and had an unremarkable neonatal course, initially presented with a two‐week history of rhinorrhea and mild cough. She remained well until one day before her first presentation, when she developed noisy breath sounds and increased work of breathing, characterized by rapid, labored breathing, perioral cyanosis, and hypoactivity; notably, she was afebrile.

**Figure 1:**
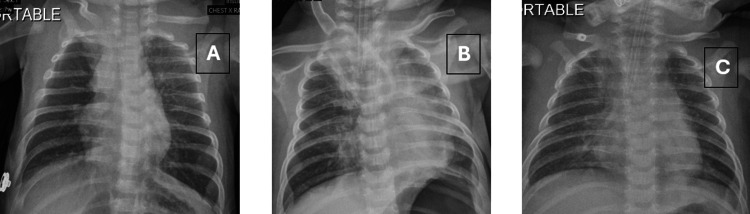
A: At admission, showing bilateral hyper‑inflated lungs and hyper‑accentuated broncho‑vascular markings, consistent with acute bronchiolitis. B: After the first intubation, showing right upper lobe collapse and left-sided opacities with appropriate endotracheal tube position. C: Before the first extubation, showing clear lung fields with satisfactory lung inflation and no consolidation or effusion.

On admission, the infant appeared sick and was in moderate-to-severe respiratory distress. Vital signs were as follows: heart rate 154 bpm, respiratory rate 68 breaths/min, blood pressure 97/55 mmHg, and oxygen saturation of 98% on HFNC at 8 L/min with an FiO₂ of 50%. She was alert (GCS 15/15) with an open, soft, and flat anterior fontanelle and preserved tone. Cardiovascular examination revealed tachycardia with normal heart sounds and no murmurs, while pulmonary examination demonstrated tachypnea with subcostal retractions, audible wheezing, and bilateral crepitations with decreased air entry. Abdominal and skin examinations were unremarkable aside from mild mottling. Initial labs were significant for mild leukocytosis WBC 15.3 × 10³/µL, with 25% neutrophils and 58.9% lymphocytes, and elevated CRP 10.5 mg/L (Table 1). A chest radiograph was obtained on admission (Figure 1A).

**Table 1:** Laboratory findings during admission WBCs: white blood cells; Hb: hemoglobin; Plt: platelets; BUN: blood urea nitrogen; CRP: C-reactive protein; LDH: lactate dehydrogenase; RBCs: red blood cells; ALT: alanine aminotransferase; AST: aspartate aminotransferase

Variable	Day 1	Day 6	Day 7	Day 8	Day 9	Day 10	Day 11	Day 14	Reference range
WBCs (× 10³/µL)	15.3	8.2	9.3	6.4	13.8	12.9	8.1	14.4	6-18
Neutrophils%	25.8	31	65.5	50	31.9	32.6	27.7	23	25-60
Lymphocytes%	58.9	45	16.9	34	56.2	57.8	59	67	20-70
Hb (g/dl)	10.9	10.1	10.7	11.2	11.3	10.3	8.9	9.9	12.5-20.5
Plt (× 10³/µL)	505	366	537	370	385	349	403	881	150-450
BUN	3.4	1.1	3.9	2.2	2.7	2.7	1.3	2.9	1.2-6
Creatinine (µmol/L)	27.6	25.3	43.6	33.9	33.5	27.7	21.2	21.9	15-37.1
CRP (mg/L)	10.5	12.4	9.6	4	5.6	29.8	28.8	1.8	0-5
Procalcitonin (ng/mL)	1.32	0.21	2.5	-	1.06	0.40	-	-	> 0.1 ng/ml, no systemic inflammatory response 0.1 – 0.49, minor or no significant inflammatory response. 0.5 – 1.99, moderate risk for progression to severe systemic infection. 2.0 – 9.99, severe systemic inflammatory response, most likely due to sepsis, unless other causes are known. ≥ 10.0, high likelihood of severe sepsis or septic shock.
pH	-	-	7.1	-	-	-	-	-	7.32-7.42
pCO2 (mmHg)	-	-	81	-	-	-	-	-	38-52
HCO3 (mmol/L)	-	-	20	-	-	-	-	-	20-26
D-dimer (ng/mL)	-	-	947	-	-	-	-	-	< 500
Serum LDH (U/l)	-	-	193	-	-	-	-	-	180-360
Pleural Protein (g/dL)	-	-	0.37	-	-	-	-	-	< 2
Pleural LDH (IU/L)	-	-	9.42	-	-	-	-	-	
Pleural RBCs (cells/µL)	-	-	100	-	-	-	-	-	
Pleural WBCs (cells/µL)	-	-	3.75	-	-	-	-	-	
ALT (IU/L)	-	-	36	40	-	-	42	49	5-55
AST (IU/L)	-	-	43	53	-	-	51	61	5-34
Albumin (g/l)	-	-	33	29	-	-	26	33	38-54
Total protein (g/l)	-	-	50	45	-	-	41	50	44-76

The infant was initially managed on HFNC with flow of 8 L/min; however, despite acceptable saturations, her respiratory distress persisted. She soon developed apnea with desaturations. Nebulized bronchodilators, chest physiotherapy, and airway suctioning were administered, and respiratory support was escalated to nCPAP (initial PEEP 7 cm H₂O, increased to 8 cm H₂O) as the FiO₂ requirement rose to 80%. A dexmedetomidine infusion was initiated to achieve sedation. Due to persistent severe distress, rising pCO₂, and high oxygen requirements, the patient was intubated. Post-intubation chest X-ray (Figure 1B) confirmed proper ETT position, right upper lobe collapse, and left-sided opacities. Sedation was maintained with fentanyl and midazolam, and dexmedetomidine was discontinued. Blood, tracheal aspirate, and urine cultures were obtained. A respiratory viral panel by polymerase chain reaction (PCR) was positive for RSV. 

Few hours later, the patient developed hypotension managed with three 0.9% sodium chloride solution boluses (15 ml/kg each) and a transient adrenaline infusion. Midazolam was held, and a low-dose ketamine infusion was started. The epinephrine infusion was discontinued a few hours later. Over the next several days, ventilator settings were gradually weaned in accordance with serial VBGs. All cultures returned negative. 

After five days of mechanical ventilation (Day 6 of admission), a chest X-ray was obtained (Figure 1C), then the infant was extubated to HFNC (8 L/min, FiO₂ 60%) with stable saturation and equal bilateral air entry.

However, six hours post-extubation, she acutely redeveloped marked respiratory distress with bilateral wheezing and prolonged expiratory phases. Despite intensive bronchodilator therapy and non-invasive ventilation, her respiratory failure recurred, necessitating re-intubation and CPAP/BiPAP trials with mild sedation; the patient’s distress persisted. VBG: pH 7.1, pCO₂ 81 mmHg, HCO₃⁻ 20 mmol/L. So, she required urgent re-intubation and mechanical ventilation with high settings.

Approximately 18 hours after re-intubation, the infant experienced recurrent bradycardia and desaturation episodes requiring bag-mask ventilation. Signs of poor perfusion and anuria (no urine output for 6 hours) were noted. Fluid resuscitation was provided with three 0.9% sodium chloride solution boluses (20 ml/kg, then 15 ml/kg twice), and a continuous epinephrine infusion was initiated. Repeat cultures (blood, urine, and tracheal aspirate) were obtained.

In the setting of ongoing clinical instability and concern for evolving sepsis despite initially negative microbiological results, empirical antimicrobial therapy was initiated with intravenous meropenem (30 mg/kg/dose every eight hours) in combination with vancomycin (15 mg/kg/dose every six hours), while a repeat respiratory viral panel by PCR remained positive for RSV.

**Figure 2:**
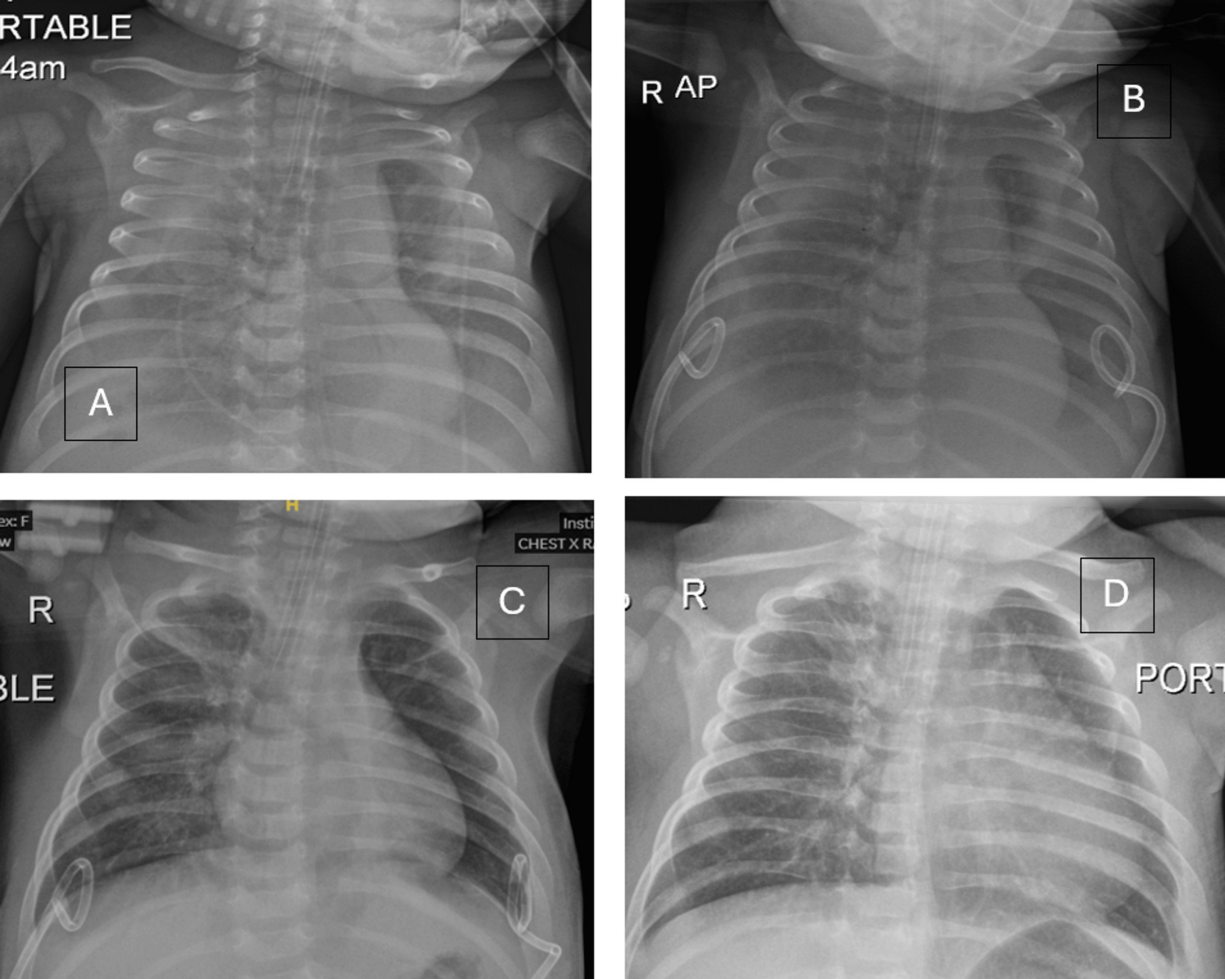
A: Chest radiograph demonstrating marked bilateral pleural effusions with blunting of both costophrenic angles and diffuse lung opacities, worse on the right. B: After insertion of bilateral pigtail catheters, showing early regression of pleural fluid but persistent reduced lung volumes and underlying collapse/consolidation. C: Before removal of pigtails, showing good lung inflation with clear costophrenic angles, indicating near‑complete resolution of effusions. D: After removal of pigtails, demonstrating maintained clear costophrenic angles bilaterally and improved aeration.

A chest radiograph at this juncture revealed bilateral lung collapse with significant pleural effusions (Figure 2A). An urgent echocardiogram demonstrated mild left ventricular hypertrophy with preserved systolic function, no pulmonary hypertension, and a small pericardial effusion. Bedside chest ultrasonography confirmed bilateral large-volume pleural effusions with internal echoes. An abdominal ultrasound revealed gallbladder wall thickening with edema and pericholecystic fluid, minimal free fluid in the right iliac fossa, and bilateral inguinal lymphadenopathy with preserved morphology.

At the time of effusion detection, creatinine had risen from 25.3 to 43.6 µmol/L, with D-dimer 947 ng/mL and procalcitonin 2.5 ng/mL, prompting concern for evolving multi-organ dysfunction and justifying escalation of invasive drainage. Ultrasound-guided bilateral pigtail catheters were inserted at the bedside (Figure 2B). To minimize the risk of re-expansion pulmonary edema, pleural fluid was aspirated in aliquots of 10 ml/kg from each side hourly over three hours, after which both pigtail catheters were connected to an underwater seal. The total drainage was 230 ml on the right and 200 ml on the left (Figure 2C). Following chest tube placement, the epinephrine infusion was discontinued, and urine output improved with additional intravenous fluids and diuretics (furosemide). Pleural fluid analysis revealed a clear, colorless fluid with a white blood cell count of 3.75 cells/µL, red blood cell count of 100 cells/µL, lactate dehydrogenase (LDH) of 9.42 IU/L, and protein of 0.37 g/dL, with corresponding serum protein of 50 g/L and LDH of 193 IU/L. The pleural fluid protein‑to‑serum protein ratio, pleural fluid LDH‑to‑serum LDH ratio, and absolute pleural LDH level were all well below the exudative thresholds defined by Light’s criteria, confirming a transudative effusion.

Cultures of pleural fluid, as well as repeat blood, urine, and tracheal aspirate cultures, remained negative. Vancomycin was discontinued after three days, and Meropenem was continued for a total of seven days. Chest tubes were removed after three days (Figure 2D), and serum creatinine returned to baseline over the next 48 hours.

**Figure 3:**
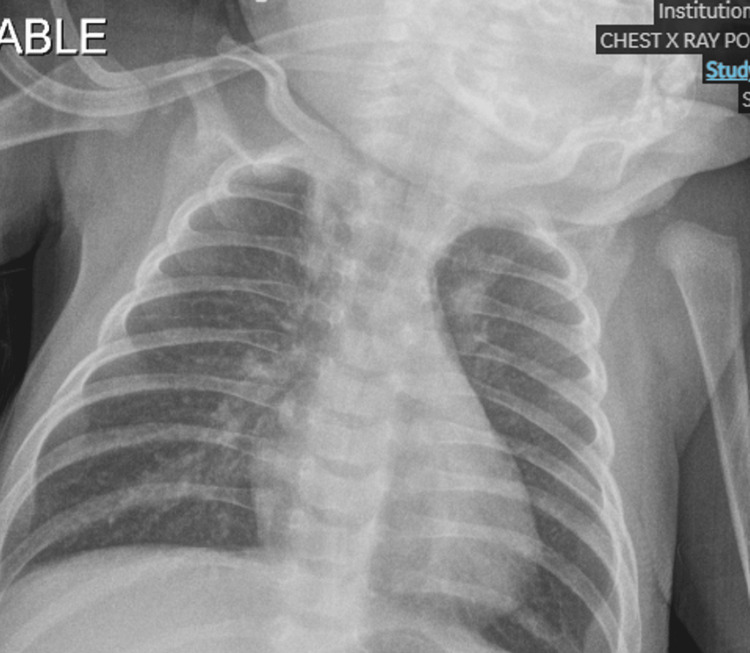
Chest radiograph after the second extubation showing well‑expanded lungs without pleural effusion or new consolidation.

**Figure 4:**
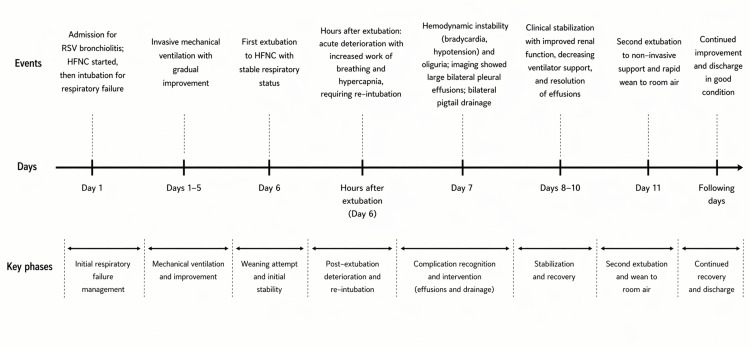
Clinical timeline of the infant with severe RSV bronchiolitis, highlighting key respiratory and hemodynamic events, onset of large bilateral pleural effusions, and timing of bilateral pigtail drainage and recovery. RSV: Respiratory syncytial virus

She was gradually weaned off ventilatory support based on serial VBGs and clinical tolerance (extubated again on day 11 of admission) and transitioned to room air over the following several days (Figure 3). Table 1 summarizes the laboratory findings during the course of admission, and Figure 4 summarizes key clinical events as a timeline.

## Discussion

This case represents a previously healthy eight-week-old infant who developed severe RSV bronchiolitis complicated by bilateral massive pleural effusions, an exceptionally rare manifestation in pediatric viral respiratory infections. The clinical course was characterized by progressive respiratory failure, cardiovascular instability, and eventual recovery following bilateral pleural drainage and intensive supportive care. The absence of bacterial pathogens in all cultures, normal echocardiography, and persistent RSV positivity strongly suggest a direct viral etiology for the pleural effusions.

Pleural effusion is a well-recognized complication of bacterial pneumonia, heart failure, and systemic diseases; however, it remains a rare complication of viral bronchiolitis, particularly in previously healthy infants [4,6]. There are scattered reports indicating that RSV alone can induce severe complications, including pneumothorax and multi-organ failure, suggesting that RSV pathophysiology is broader and potentially more severe than previously appreciated, even in the absence of comorbidities [7,8], which is more consistent with the case of our patient.

The precise pathophysiological mechanisms underlying the development of pleural effusions in viral bronchiolitis remain incompletely understood; however, several plausible hypotheses have been proposed. Increasing evidence supports a cytokine-mediated inflammatory pathway [5]. RSV infection induces a robust proinflammatory response characterized by elevated levels of key cytokines, including interleukin (IL)-1β, IL-6, IL-8, tumor necrosis factor-α (TNF-α), and interferon-γ (IFN-γ) [9, 10]. Studies in RSV-infected infants demonstrate significantly increased concentrations of these mediators in respiratory secretions compared to healthy controls and an association with poor prognosis [11,12]. Animal studies of viral pericarditis have demonstrated that inflammatory injury accompanied by markedly elevated IL-1β and IL-6 in serosal fluid correlates with increased serosal permeability [13]. Given RSV's documented ability to upregulate these same mediators, it is plausible that RSV-associated pleural effusions arise through similar cytokine-driven mechanisms that increase capillary permeability in both visceral and parietal pleura [14]. Further studies are needed to evaluate cytokine levels in pleural fluid in cases of pleural effusion associated with viral bronchiolitis.

In our patient, the development of a transudative pleural effusion, rather than the exudative pattern typically expected with viral inflammation, likely reflects multifactorial pathophysiologic processes observed in critically ill, mechanically ventilated infants with RSV bronchiolitis [14,15]. Aggressive fluid resuscitation to manage hypotension, compounded by elevated central venous pressure from mechanical ventilation, increases the hydrostatic pressure within the systemic capillaries of the parietal pleura, promoting fluid movement into the pleural space. The use of high PEEP further contributed by elevating pleural pressure, reducing lymphatic drainage efficiency, and compressing the thoracic duct, all of which diminish fluid clearance from the pleural space. Additionally, the presence of lung collapse and atelectasis on imaging may have enhanced localized negative pleural pressure, facilitating further fluid accumulation via the “trapped lung” effect, commonly seen in mechanically ventilated patients with poor lung compliance [15,16]. Mechanical ventilation can be challenging in patients with heterogeneous lung disease like severe RSV bronchiolitis [17]. The critical opening pressure is elevated in a non-uniform manner, making optimal PEEP selection challenging and potentially contributing to regional lung collapse and pleural complications [18].

The transudative nature of the effusion in our patient would suggest that in critically ill infants with RSV and mechanical ventilation, systemic and mechanical factors, rather than local inflammatory responses, may dominate in pleural effusion formation. Pleural fluid analyses are still lacking to confirm the protein content in the literature, and all reported cases have relied on ultrasound findings without studying pleural fluid characteristics directly [5].

A landmark study of 69 infants with acute bronchiolitis revealed pleural effusion in 49.2% of cases, detected via thoracic ultrasonography, regardless of disease severity. These effusions, typically small and asymptomatic, showed no significant correlation with clinical outcomes or recovery timelines. The study population included both non-ventilated patients and those with severe bronchiolitis, though mechanical ventilation status was not explicitly analyzed as an independent variable. The high prevalence suggests that pleural effusion constitutes a common but underrecognized feature of viral bronchiolitis pathophysiology. The presence of pleural effusion was significantly more common in patients with underlying risk factors. These findings suggest that while pleural effusion may occur more often than expected in bronchiolitis, clinically significant or symptomatic effusions remain rare and may be limited to those with severe disease or predisposing conditions [5].

The application of lung ultrasonography in viral bronchiolitis has emerged as a revolutionary diagnostic and prognostic method, with rising data supporting its integration into clinical practice while arguing certain limits [19,20]. Given the low overall incidence of clinically significant pleural effusion in RSV bronchiolitis, routine imaging for all patients may not be justified. However, in cases where patients show signs of severe disease or deteriorating cardiopulmonary status, targeted screening using modalities such as chest ultrasound could be beneficial. Current clinical guidelines do not universally recommend such screening, reflecting a gap in our understanding of which patients may benefit most from early detection and intervention. Further research is warranted to determine whether a more proactive imaging strategy could improve outcomes in severe cases of RSV bronchiolitis.

## Conclusions

Our case highlights that severe RSV bronchiolitis in previously healthy infants can be complicated by massive bilateral pleural effusions with a transudative profile, likely driven by hemodynamic stress, fluid resuscitation, and positive‑pressure ventilation rather than primary viral pleuritis. This underscores the importance of considering pleural fluid accumulation as a potential contributor to cardiorespiratory instability in critically ill, ventilated infants with RSV, and of integrating pleural‑effusion assessment into the management of such patients. Further case series and mechanistic studies are needed to clarify the incidence, triggers, and optimal management of pleural effusions in mechanically ventilated children with RSV bronchiolitis, which may ultimately refine ventilator strategies, fluid management, and monitoring protocols in this vulnerable population.
